# Pairwise shared genomic segment analysis in high-risk pedigrees: application to Genetic Analysis Workshop 17 exome-sequencing SNP data

**DOI:** 10.1186/1753-6561-5-S9-S9

**Published:** 2011-11-29

**Authors:** Zheng Cai, Stacey Knight, Alun Thomas, Nicola J Camp

**Affiliations:** 1 Department of Biomedical Informatics, University of Utah, 391 Chipeta Way, Salt Lake City, UT 84108, USA; 2 Division of Genetic Epidemiology, Department of Internal Medicine, University of Utah, 391 Chipeta Way, Salt Lake City, UT 84108, USA

## Abstract

We applied our method of pairwise shared genomic segment (pSGS) analysis to high-risk pedigrees identified from the Genetic Analysis Workshop 17 (GAW17) mini-exome sequencing data set. The original shared genomic segment method focused on identifying regions shared by all case subjects in a pedigree; thus it can be sensitive to sporadic cases. Our new method examines sharing among all pairs of case subjects in a high-risk pedigree and then uses the mean sharing as the test statistic; in addition, the significance is assessed empirically based on the pedigree structure and linkage disequilibrium pattern of the single-nucleotide polymorphisms. Using all GAW17 replicates, we identified 18 unilineal high-risk pedigrees that contained excess disease (*p* < 0.01) and at least 15 meioses between case subjects. Eighteen rare causal variants were polymorphic in this set of pedigrees. Based on a significance threshold of 0.001, 72.2% (13/18) of these pedigrees were successfully identified with at least one region that contains a true causal variant. The regions identified included 4 of the possible 18 polymorphic causal variants. On average, 1.1 true positives and 1.7 false positives were identified per pedigree. In conclusion, we have demonstrated the potential of our new pSGS method for localizing rare disease causal variants in common disease using high-risk pedigrees and exome sequence data.

## Background

The original shared genomic segment (SGS) method is an analytical technique designed specifically for finding rare disease variants in high-risk extended pedigrees [1,2]. It was designed for use with dense single-nucleotide polymorphism (SNP) genotyping to identify regions of identical-by-state (IBS) sharing between multiple distantly related case subjects. Identical-by-descent (IBD) sharing between distant relatives is increasingly improbable with genetic distance and is expected to be short. Regions IBD must also be IBS. IBS sharing is much simpler to identify, and long lengths of IBS sharing that are not expected by chance are likely to be shared IBD. Therefore the identification of long, shared IBS regions among distant case subjects in a family may identify the area that is also shared IBD; this IBD area contains genes that predispose the individual to a given disease trait.

For rare diseases (≤ 1% frequency in the population) with rare causal variants (minor allele frequency [MAF] ≤ 0.005), the original SGS method based on sharing among all case subjects in high-risk pedigrees has good power, even under moderate penetrance. Under these scenarios, the likelihood of intrafamilial heterogeneity is decreased and hence the likelihood that all case subjects share the same causal variant is increased. However, for more common diseases, where not all case subjects may be affected by the same underlying causal variants, an SGS method based on sharing of all case subjects may lack power. Instead, examining sharing among all pairs of case subjects would increase the robustness of the method.

In this study, we develop a new pairwise SGS method that examines the sharing among all pairs of case subjects. We apply the pairwise SGS (pSGS) method to a set of 18 high-risk pedigrees from the Genetic Analysis Workshop 17 (GAW17) data set. The known population disease prevalence rate for the simulated disease is about 30%.

## Methods

**Figure 1:**
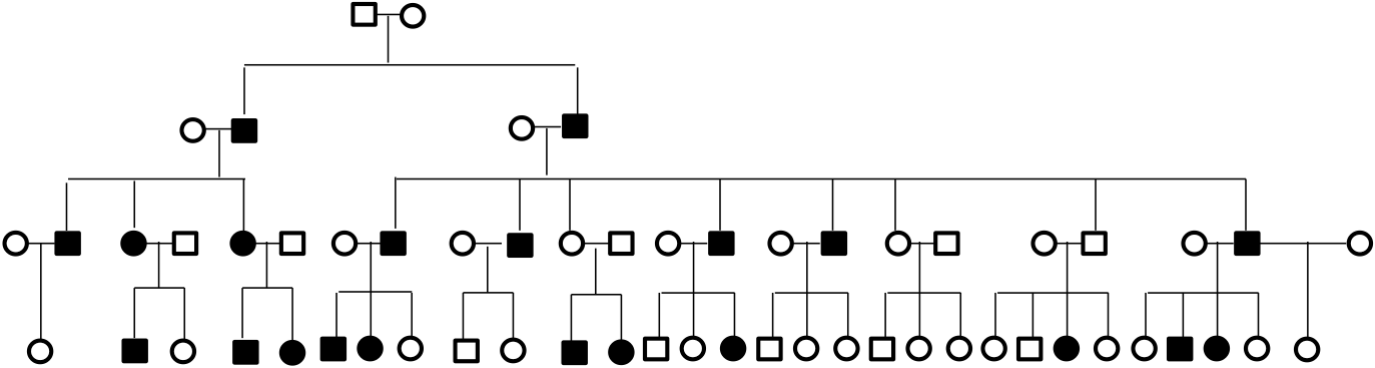
**Pedigree structure.** Case status shown for replicate 15.

Each replicate of the GAW17 family data set consists of eight different pedigree structures, each with four generations. We split the pedigrees into unilineal lines of descent, which include two common ancestral founders along with their descendants. We use all 200 replicates and identify high-risk pedigrees, defined as those with at least 15 total meioses between case subjects and a statistical excess of disease (*p* < 0.01). We selected the threshold of at least 15 meioses because the genome-wide probability of IBD sharing by chance across 15 meioses is approximately 0.05 [1]. Eighteen pedigrees meet the criteria of 15 total meioses and excess disease. All these pedigrees are derived from GAW17 pedigree structure 7, which was based on a unilineal descendants from founders 7010 and 7013. This pedigree structure contains 59 individuals, with 16 founders and 43 related descendants (Figure 1). Although the structure is consistent, each of the 18 pedigrees contains a different set of case subjects, according to the GAW17 disease simulation. The average number of related cases is 21.72, with a range of 21–24 (*p*-values for excess disease range from 0.002 to 0.007). Within each of these 18 extended high-risk pedigrees, there are 18 rare causal variants that are polymorphic (from more than 100 causal variants in the full disease model).

For each pedigree, we perform a genome-wide pSGS analysis by examining the average sharing across all pairs of case subjects. The test statistic for the pSGS analysis (SH^case^(*k*)) is similar to that previously proposed by Nolte et al. [3] for a case-control haplotype-sharing method and is defined as:(1)

where *R_ij_* is the run length, defined as the number of SNPs in a row that are sharing IBS. *R_ij_* is obtained from a pairwise comparison between case subject *i* and case subject *j* at locus *k*.

Statistical significance is determined using an empirical approach based on a hidden Markov model. To ensure that an appropriate linkage disequilibrium (LD) pattern is preserved in the empirical approach, we estimate the LD model using graphical models [4]. The genotypes of 78 independent individuals from the GAW17 family data, including the 16 founders in the pedigree, are used for LD model estimation. Subsequently, we use a gene-drop program [5] to assign haplotypes to founders based on the estimated LD model and to simulate genotypes for descendants based on a genetic map according to the known SNP base-pair position and the law of Mendelian segregation, without regard to the disease status (i.e., under the null hypothesis *H*_0_). Using these simulated genotype configurations, we calculate a null pSGS statistic for each marker position in the genome. Significance of each marker’s pSGS value is assessed against an empirical distribution of at least 10,000 null test statistics for that same position.

We use a significance threshold of *α* = 0.001 because this level appears to differentiate peaks from background signal. Pairwise shared genomic segments that reached the significance threshold are identified, and true-positive and false-positive rates are calculated based on the answers. A true positive is defined as a region containing a causal gene where the actual causal variant is polymorphic in the pedigree cases. For each true positive, we summarize the frequency of the causal variant in the pedigree case subjects and compare it to the population frequency for the variant.

## Results

In 13 out of the 18 pedigree replicates (72.2%) we were successful in identifying at least one region that contained a true and polymorphic causal variant. Based on this estimate, two pedigrees would gain more than 90% power to identify a genomic region containing at least one true causal locus. Using a significance threshold of 0.001 and two high-risk pedigrees, one would expect to identify an average of 5.5 regions including 2.1 true positives and 3.4 false positives.

Table 1 shows the true causal variants found in each pedigree. Four (22%) out of the 18 rare causal variants that were polymorphic in our selected pedigrees were identified by the pSGS method. The most frequently identified causal variants were SNPs C9S444, which was found in 9 of 18 replicates (50%), C1S9189 (28%), and C10S2109 (22%). Variant C4S4935 on chromosome 4 was found only once; however, this variant was carried by more than 90% of the case subjects in the pedigree, compared to a frequency of less than 0.1% in the population (the largest discrepancy in frequency identified). Five replicates identified no true positives, nine replicates identified one, three replicates identified two, and one pedigree identified four.

**Table 1:** Significant true-positive genes by pedigree found using PSGS analysis

Replicate	Chromosome	*p*-value	Causal gene	Causal SNP	Frequency in cases (%)	Frequency in population (%)	Run length range in causal gene
25	1	1 × 10^–4^	*PIK3C2B*	C1S9189	4.35	0.65	1,152.577–1,192.443
	9	1 × 10^–5^	*VLDLR*	C9S444	17.39	0.14	793.933–802.051
27	9	1 × 10^–4^	*VLDLR*	C9S444	14.29	0.14	798.038–816.491
29	1	0.001	*PIK3C2B*	C1S9189	4.76	0.65	1,065.976–1,106.357
	4	0.001	*VEGFC*	C4S4935	90.48	0.07	639.6238
	9	1 × 10^–6^	*VLDLR*	C9S444	9.52	0.14	801.038–809.448
	10	0.001	*SIRT1*	C10S3109	61.90	0.07	870.229–870.229
49	10	0.001	*SIRT1*	C10S3109	56.52	0.07	919.134–919.134
75	1	2 × 10^–4^	*PIK3C2B*	C1S9189	4.76	0.65	1,127.395–1,159.105
85	1	2 × 10^–4^	*PIK3C2B*	C1S9189	4.35	0.65	1,159.779–1,188.854
	9	1 × 10^–4^	*VLDLR*	C9S444	13.04	0.14	764.763–770.269
94	1	1 × 10^–4^	*PIK3C2B*	C1S9189	4.76	0.55	1,232.038–1,256.771
116	9	4 × 10^–4^	*VLDLR*	C9S444	13.64	0.14	772.983–776.619
129	9	5 × 10^–4^	*VLDLR*	C9S444	20.83	0.14	712.761–728.337
142	9	9 × 10^–4^	*VLDLR*	C9S444	19.05	0.14	708.8238–711.8381
	10	3 × 10^–4^	*SIRT1*	C10S3109	57.14	0.07	863.629–863.629
184	9	7 × 10^–4^	*VLDLR*	C9S444	23.81	0.14	734.410–750.419
190	9	4 × 10^–5^	*VLDLR*	C9S444	18.18	0.14	779.281–782.654
199	10	6 × 10^–4^	*SIRT1*	C10S3109	71.43	0.07	984.443–984.443

Replicates 15, 101, 144, 182, and 200 did not identify any true-positive genes. Significance threshold = 0.001. Frequencies of causal SNPs in case subjects were calculated from 18 pedigree replicates.

It is interesting to note that 10 out of 18 replicates identified the region on chromosome 19 (*p* ≤ 0.001) that contains causal genes *HIF3A* and *RRAS*. However, the case subjects in our pedigree replicates were not polymorphic for the risk variants in these genes, and we classified this finding as a false positive.

## Discussion

We successfully localized four true-positive causal SNPs from the 18 disease-susceptible SNPs that were polymorphic in our pedigree replicates. Additional genes, such as *VNN3* on chromosome 6, would have been identified at lower significance thresholds (*p* ≤ 0.01), but this would also have increased false-positive findings. The inability to detect the other rare variants may have several explanations. First, we had a small sample size of only 18 high-risk pedigree replicates, and our results may not adequately reflect what can and cannot be identified. Second, all GAW17 replicates were generated from the same genotype data, and because selected replicates in our study were all derived from the same structure, the genotype data were consistent across all 18 replicates. Even though the case status of individuals changed across replicates, the underlying correlation may limit the scope of which variants the pedigrees would have the power to detect. Third, we restricted our pedigrees to be those with a statistically significant excess of case subjects; hence we may have also increased sporadic cases (heterogeneity). Finally, our pSGS statistic disregards the specific relationships between each pair of case subjects, which may not be optimal for power. To investigate this, we considered a weighted mean based on the number of meioses separating each pair. Some improvement was observed, but it did not qualitatively alter the results here (data not shown).

## Conclusions

Identifying rare risk variants in common diseases is still a challenge. We used the GAW17 mini-exome family data set to investigate the utility of a novel pairwise SGS method for detecting rare variants in a common trait (about 30% prevalence). Our results, based on 18 extended high-risk pedigree replicates, suggest that as few as two pedigrees may offer greater than 90% power to detect at least one true causal rare variant. Because few pedigrees are needed and only case subjects are studied, the method requires minimal genotyping or sequencing to achieve the power and hence is cost-efficient.

It is important to balance power and false-positive findings. Here we estimate a ratio of 1.1:1.7 true:false positive findings per genome based on a significance threshold of 0.001. We believe that this is a reasonable ratio for a gene discovery study. Nonetheless, exploring avenues to increase power and reduce false positives is a useful endeavor. As mentioned, more sophisticated weighting statistics may increase power. Alternative significance thresholds may be superior for balancing the true and false positives. For example, a threshold of 1 × 10^−5^ would have resulted in two true positives and one false positive across the 18 pedigree replicates (0.15:0.55 true:false positive findings per genome); however, this more stringent threshold would necessitate at least 14 pedigree replicates for at least 80% power. Finally, a requirement of matching the same region across multiple pedigree replicates may also help the true:false ratio, although these GAW17 data are not ideal for exploring such strategies because of the way the data were generated and this also may not be fruitful if the underlying risk variants are truly rare. In conclusion, our new pairwise SGS method has demonstrated good potential for detecting rare variants in a common disease using few high-risk pedigrees. Future work will involve additional steps to improve the method, in order to balance true- and false-positive findings.

## Competing interests

The authors declare that there is no conflict of interests.

## Authors’ contributions

All authors contributed to the study design and approved the final manuscript. SK carried out the quality control for the dataset. ZC performed the statistical analysis. All authors participated in the drafting of the manuscript.
